# Efficacy and Safety of 4-Month Rifapentine-Based Tuberculosis Treatments in Persons with Diabetes

**DOI:** 10.3201/eid3103.241634

**Published:** 2025-03

**Authors:** Ekaterina V. Kurbatova, William C. Whitworth, Lakshmi Praveena Peddareddy, Patrick P.J. Phillips, Nigel A. Scott, Kia E. Bryant, Rodney Dawson, Sandra Wagner Cardoso, Wadzanai Samaneka, Melissa Engle, Ziyaad Waja, Erin Sizemore, Wendy Carr, Kelly E. Dooley, Radojka Savic, Susan Swindells, Richard E. Chaisson, Susan E. Dorman, Payam Nahid, Nhung V. Nguyen, AIDS Clinical Trials Group A5349,, Tuberculosis Trials Consortium Study 31

**Affiliations:** Centers for Disease Control and Prevention, Atlanta, Georgia, USA (E.V. Kurbatova, W.C. Whitworth, L.P. Peddareddy, N.A. Scott, K.E. Bryant, E. Sizemore, W. Carr); University of California– San Francisco Center for Tuberculosis, San Francisco, California, USA (P.P.J. Phillips, R. Savic, P. Nahid); University of Cape Town Lung Institute, Cape Town, South Africa (R. Dawson); Fundação Oswaldo Cruz Insituto Nacional de Infectologia Evandro Chagas, Rio de Janeiro, Brazil (S. Wagner Cardoso); Milton Park Clinical Research Site, Harare, Zimbabwe (W. Samaneka); Audie L. Murphy Veterans Administration Medical Center, San Antonio, Texas, USA (M. Engle); Wits Health Consortium Perinatal HIV Research Unit, Johannesburg, South Africa (Z. Waja); Vanderbilt University Medical Center, Nashville, Tennessee, USA (K.E. Dooley); University of Nebraska Medical Center, Omaha, Nebraska, USA (S. Swindells); Johns Hopkins University School of Medicine, Baltimore, Maryland, USA (R.E. Chaisson); Medical University of South Carolina, Charleston, South Carolina, USA (S.E. Dorman); Vietnam National Tuberculosis Program/University of California–San Francisco Research Collaboration Unit, Hanoi, Vietnam (N.V. Nguyen)

**Keywords:** tuberculosis and other mycobacteria, diabetes, phase 3 clinical trial, rifapentine, moxifloxacin, antimicrobial resistance, respiratory infections, bacteria, United States

## Abstract

A previous study demonstrated noninferior efficacy of 4-month rifapentine/moxifloxacin regimen for tuberculosis (TB) treatment compared with the standard regimen. We explored results among study participants with diabetes. Among 2,516 randomized participants, 181 (7.2%) had diabetes. Of 166 participants with diabetes in the microbiologically eligible analysis group, 26.3% (15/57) had unfavorable outcomes in the control regimen, 13.8% (8/58) in the rifapentine/moxifloxacin regimen, and 29.4% (15/51) in the rifapentine regimen. The difference in proportion of unfavorable outcomes between the control and rifapentine/moxifloxacin arms in the microbiologically eligible analysis group was –12.5% (95% CI –27.0% to 1.9%); the difference between the control and rifapentine arms was 3.1% (95% CI –13.8% to 20.0%). Safety outcomes were similar in the rifapentine/moxifloxacin regimen and control arms. Among participants with TB and diabetes, the rifapentine/moxifloxacin arm had fewest unfavorable outcomes and was safe. Our findings indicate that the rifapentine/moxifloxacin regimen can be used in persons with TB and diabetes.

Tuberculosis (TB) and diabetes are important public health concerns because they have high global prevalence and high mortality rates (*1*). The presence of diabetes in patients with TB has been shown to be associated with poor TB treatment outcomes, such as prolonged times for sputum smear or sputum culture conversion, treatment failure, relapse, and an increased mortality rate (*2*–*11*). Worse treatment outcomes in persons with diabetes might be attributable to several interwoven factors, including immune dysregulation, lower drug exposures, and higher frequency of underlying conditions (*12*–*14*).

The Tuberculosis Trials Consortium Study 31/AIDS Clinical Trials Group A5349 (https://clinicaltrials.gov/study/NCT02410772) was a randomized, controlled, noninferiority phase 3 trial that examined two 4-month treatment-shortening rifapentine-containing regimens compared with the standard 6-month control regimen for treatment of drug-susceptible pulmonary TB in adults and adolescents (*15*). One investigational regimen contained rifapentine, moxifloxacin, and isoniazid administered for 4 months plus pyrazinamide administered during the first 2 months (rifapentine/moxifloxacin regimen). The other investigational regimen contained rifapentine plus isoniazid administered for 4 months plus pyrazinamide and ethambutol administered during the first 2 months (rifapentine regimen). The trial demonstrated that the 4-month rifapentine/moxifloxacin regimen had efficacy that was noninferior to that of the control and was safe and well-tolerated. The rifapentine regimen did not meet the noninferiority criteria for efficacy. In that study, the time to stable sputum culture conversion to negative was shorter in participants treated with each of the investigational 4-month regimens compared with the control regimen (*15*).

On the basis of the trial results, the rifapentine/moxifloxacin regimen has been recommended by the World Health Organization (WHO) and Centers for Disease Control and Prevention (CDC) for use for the treatment of drug-susceptible pulmonary tuberculosis (*16*,*17*). Given the importance of the TB and diabetes syndemic, we compared the efficacy and safety across study regimens for the subgroup of participants with diabetes.

## Methods

### Study Design, Participant Enrollment, Randomization, and Follow-up

Full details of the parent study design, eligibility criteria, enrollment and randomization, safety monitoring, and study outcomes have been previously published (*15*). In brief, we enrolled participants >12 years of age with newly diagnosed pulmonary TB during January 2016–October 2018. We randomly assigned enrolled participants in a 1:1:1 ratio to 1 of the 3 regimens (i.e., control, rifapentine, or rifapentine/moxifloxacin). We administered study drugs once daily, by directly observed therapy, on >5 of 7 days/week.

The study protocol required diabetes screening before enrollment. Hemoglobin A1c (HgbA1c) was the preferred test. If such testing was not available, we collected readings of fasting blood glucose (defined as no caloric intake for >8 hours) or nonfasting blood glucose. A prior diagnosis of diabetes at the time of TB diagnosis was self-reported by the study participants and verified with medical documentation when available. Concomitant medications taken during the study were routinely recorded by the study sites on the concomitant medications case report form and thereafter coded and characterized by using the WHO Drug Dictionary’s anatomic therapeutic classification system (*18*). We used the WHO Drug Dictionary’s standardized drug groupings to identify class 2 category drugs used in diabetes (*18*).

Because we used different approaches in different sites for capturing data on diabetes, we developed a consensus definition of diabetes. We classified participants as having diabetes if any of the following case selection criteria were met at baseline: a prior diagnosis of diabetes, receipt of insulin or any other diabetes medications, HgbA1c >6.5%, fasting blood glucose >126 mg/dL, or nonfasting blood glucose ≥200 mg/dL.

All participants had study visits at baseline, at weeks 2, 4, 8, 12, 17, 22, and 26, and at months 9, 12, 15, and 18 after randomization (*15*). During study visits, we evaluated participants for adverse events, collected blood samples for complete blood count and biochemical analyses through week 22, and collected sputum samples for mycobacterial culture through the follow up. We collected adverse event reports through the 18 months of the study follow-up period. We graded adverse events severity on the basis of Common Terminology Criteria for Adverse Events criteria version 4.03 (*19*).

The study was approved by the CDC institutional review board. Each participating institution provided for the review and approval of protocol and its informed consent documents by a local institutional or ethics committee or relied formally on the CDC institutional review board’s approval. All participants provided written informed consent. The study data were monitored by a data safety monitoring board coordinated by the study sponsor.

### Definitions of Outcomes

The primary efficacy outcome was TB disease–free survival 12 months after randomization. For each participant, we assigned a primary outcome status of favorable, unfavorable, or not assessable, as described previously; we further classified unfavorable outcomes as TB-related or not TB-related (*15*). We defined time to stable culture conversion as the time to the first of 2 consecutive negative sputum cultures without an intervening positive culture.

The primary safety outcome was the proportion of participants with grade >3 adverse events during treatment (with onset up to 14 days after the last dose of study medication). Tolerability was a secondary safety outcome and was defined as premature discontinuation of the assigned regimen for any reason other than microbiologic ineligibility.

### Analysis Populations

The microbiologically eligible analysis population included participants with culture-confirmed TB without resistance to isoniazid, rifampin, and fluoroquinolones. The assessable analysis population excluded those without an assessable outcome. We considered microbiologically eligible and assessable as primary analysis populations. Secondary analysis populations included participants who completed >75% and >95% of treatment doses (2 per protocol analysis populations), and all participants randomized (intention to treat). We included all randomized participants that started study treatment in safety analyses.

### Statistical Analysis

We used descriptive statistics to summarize the demographic and clinical characteristics among participants with diabetes. For primary efficacy and safety secondary subgroup analysis, we calculated the risk difference between the regimens and their respective 95% CIs.

### Pharmacokinetics

We sampled all participants who underwent randomization for pharmacokinetic analysis. All participants had 1–3 sparse pharmacokinetic samples (timepoints were at 0.5, 5, and 24 hours postdose), and at some sites participants had 7 intensive pharmacokinetic sampling (timepoints were at 0.5, 3, 5, 9, 12, and 24 hours postdose), conducted during weeks 2–8 of TB treatment. We determined plasma concentrations of rifapentine, 25-desacetyl-rifapentine, rifampin, isoniazid, pyrazinamide, ethambutol, and moxifloxacin by using validated high-performance liquid chromatography assays. We developed population pharmacokinetic models for each of the 6 drugs, and we calculated the individual area under the concentration time curve from 0–24 hours (AUC_0–24h_) and maximal plasma concentration (C_max_) for each drug (*20*). We compared AUC_0–24h_ and C_max_ for each drug by using t-tests by diabetes.

## Results

### Study Population

Of 2,516 randomized participants in the full study, 181 (7.2%) we classified as having diabetes (Table 1; Appendix Figure). Among 181 participants who were classified as having diabetes, 83 (45.8%) reported a prior diabetes diagnosis at enrollment. Participants with diabetes were from study sites in 12 countries (Brazil, Haiti, India, Kenya, Malawi, Peru, South Africa, Thailand, Uganda, United States, Vietnam, and Zimbabwe). The percentage of participants with diabetes among the enrolled sites was 19.3% (17/88) in sites located in South America, 15.5% (45/290) in Asia, 5.7% (104/1832) in Africa, and 4.9% (15/306) in North America.

**Table 1:** Diabetes status of 181 participants at enrollment, by tuberculosis drug regimen, in a study assessing efficacy and safety of 4-month rifapentine-based tuberculosis treatments in persons with diabetes at sites in 12 countries,* January 2016–October 2018

Criterion†	No. patients (%)			
	Control, n = 59	Rifapentine/moxifloxacin, n = 66	Rifapentine, n = 56	Total, N = 181
Hemoglobin A1c >6.5%	49 (83.1)	43 (65.2)	43 (76.8)	135 (74.6)
Prior reported diagnosis of diabetes	31 (52.5)	36 (54.5)	16 (28.6)	83 (45.9)
Receiving antidiabetic drugs‡	22 (37.3)	29 (43.9)	11 (19.6)	62 (34.3)
Fasting blood glucose >126 mg/dL	14 (23.7)	18 (27.3)	13 (23.2)	45 (24.9)
Nonfasting blood glucose >200 mg/dL	5 (8.5)	13 (19.7)	4 (7.1)	22 (12.2)

*Brazil, Haiti, India, Kenya, Malawi, Peru, South Africa, Thailand, Uganda, United States, Vietnam, and Zimbabwe. †Diabetes criteria were assessed at enrollment (baseline). Randomized trial participants meeting >1 of these criteria at enrollment were included in these analyses. ‡World Health Organization Drug Dictionary’s standardized drug groupings were used to identify class 2 category drugs used in diabetes (*18*).

We examined baseline demographics and clinical characteristics of participants with diabetes by regimen (Appendix Table 1). Overall, 67.4% were male and 32.6% female, the median age was 46 years, 16 (8.8%) participants were HIV-positive, 132 (72.9%) had baseline cavitation on chest radiograph, and the median body mass index was 21 kg/m^2^. A total of 146 (80.1%) of 181 participants had available HgbA1c results at baseline (median value 7%). Sixty-two participants (34.3%) reported receiving medications for diabetes. Among the 83 participants reporting a prior diagnosis of diabetes at baseline, 8 (9.6%) reported having diabetes mellitus type 1 and 73 (88.0%) reported having diabetes mellitus type 2; for 2 (2.4%), the type of diabetes was unknown. Twenty participants were classified as having diabetes on the basis of blood glucose test results only.

Compared with participants without diabetes, participants with diabetes were older (median age 46 vs. 30 years); more often reported Asian race (25.9% vs. 10.3%), White race (8.4% vs. 1.0%), >1 race (18.1% vs. 13.0%), or Hispanic ethnicity (13.3% vs. 2.4%); were more often enrolled at study sites in Asia (25.9% vs. 10.1%) or South America (9.6% vs. 3.1%); had higher bodyweight (56 vs. 53 kg); had smaller (<4 cm) cavity size (44.6% vs. 32.2%); and had lower (negative to 1+) smear positivity grade (55.4% vs. 42.2%) (all p<0.005) (Appendix Table 2). The presence of baseline cavitation on chest radiograph was similar (72.9% of participants with diabetes had cavitary disease vs. 72.3% participants without diabetes). We observed a shorter time-to-detection in liquid media in participants with diabetes compared with those without diabetes (8.27 days vs. 8.82 days; p = 0.03).

### Efficacy

**Figure 1:**
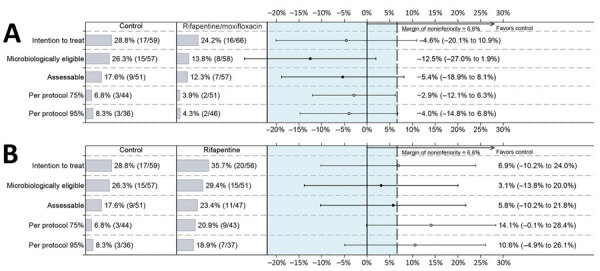
Unadjusted differences in unfavorable outcomes in each analysis population among participants with diabetes in a study assessing efficacy and safety of 4-month rifapentine-based tuberculosis treatments in persons with diabetes at sites in 12 countries (Brazil, Haiti, India, Kenya, Malawi, Peru, South Africa, Thailand, Uganda, United States, Vietnam, and Zimbabwe), January 2016–October 2018. Results of the efficacy results in all 5 analysis populations are shown: rifapentine/moxifloxacin regimen versus control regimen (A) and rifapentine regimen versus control regimen (B). Solid dots indicate primary results, open dots indicate secondary results, and error bars indicate 95% CIs. Dashed vertical line indicates the noninferiority margin of 6.6% for overall results in the randomized trial (*18*).

We included 166 participants with diabetes (91.7%) in the microbiologically eligible analysis population and 155 (85.6%) participants in the assessable analysis population (Figure 1; Appendix Table 3). Among participants in the microbiologically eligible population, unfavorable outcomes occurred in 26.3% of participants in the control regimen and 13.8% of participants in the rifapentine/moxifloxacin regimen, indicating a risk difference from control of –12.5% (95% CI –27.0% to 1.9%). Unfavorable outcomes occurred in 29.4% of participants in the rifapentine regimen, indicating a risk difference from control of 3.1% (95% CI –13.8% to 20.0%). For the assessable analysis population, unfavorable outcomes occurred in 17.6% of participants in the control regimen and 12.3% of participants in the rifapentine/moxifloxacin regimen, indicating an absolute difference from control of –5.4% (95% CI –18.9% to 8.1%). Unfavorable outcomes occurred in 23.4% of participants in the rifapentine regimen, indicating an absolute risk difference from control of 5.8% (95% CI –10.2% to 21.8%). The percentage of participants with TB-related unfavorable outcome was 5.3% in the control arm, 3.4% in the rifapentine/moxifloxacin regimen, and 19.6% in the rifapentine regimen in the microbiologically eligible population and 5.9% in the control arm, 3.5% in the rifapentine/moxifloxacin regimen, and 21.3% in the rifapentine regimen in the assessable population (Appendix Table 3). We observed no cases of acquired TB drug resistance in participants with diabetes.

In sensitivity analysis limited to the 83 participants with prior diabetes diagnosis, proportions of unfavorable outcome were slightly higher than in analysis of all participants classified as having diabetes, but differences between regimens were similar (Appendix Table 4). Participants with diabetes had higher overall proportion of unfavorable outcomes compared with participants without diabetes (microbiologically eligible population, 22.9% vs. 15.4%; assessable population, 17.4% vs. 11.4%).

### Time to Culture Conversion

**Figure 2:**
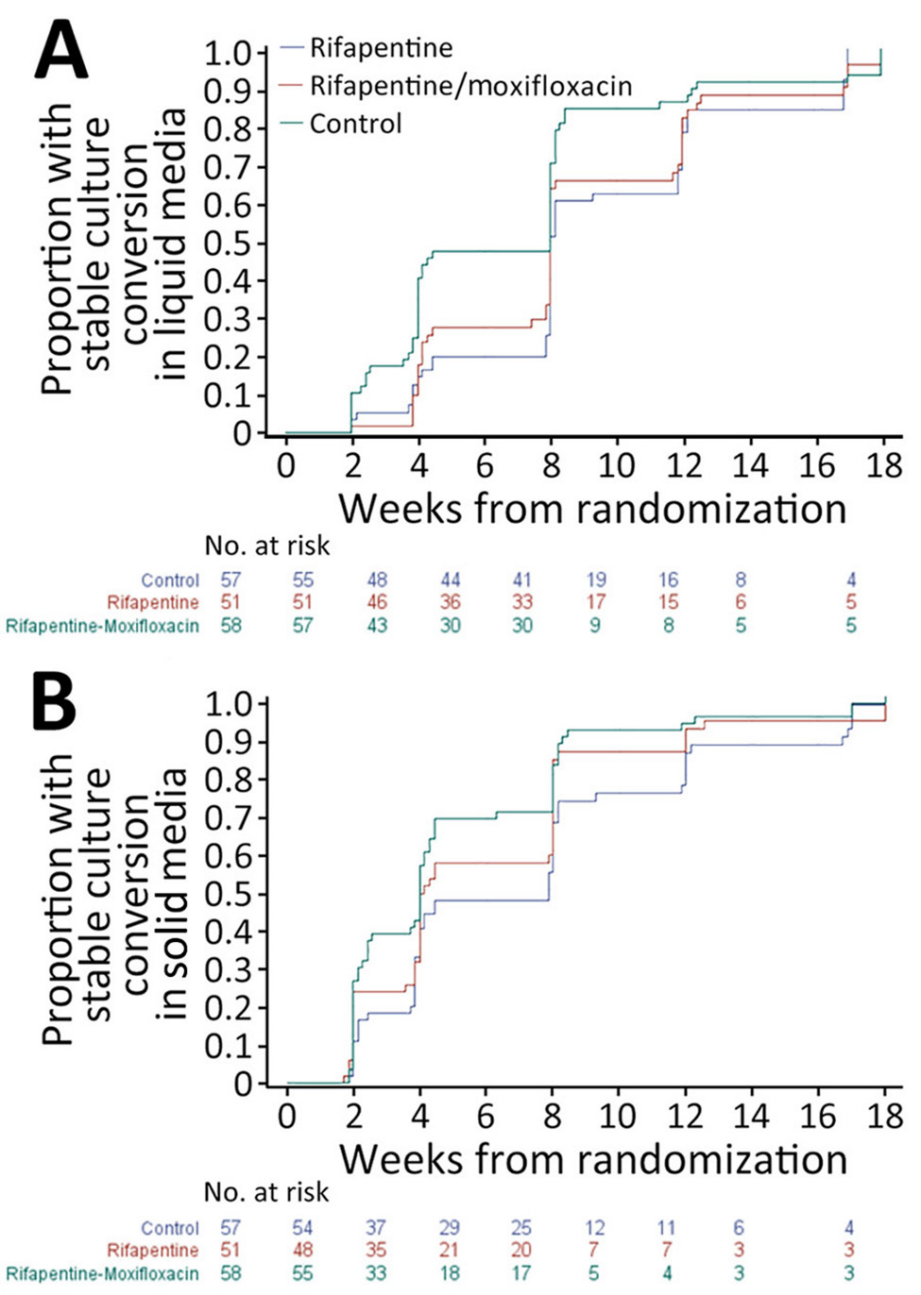
Analysis of time to sputum culture conversion (number of weeks from randomization) in liquid (A) and solid media (B) among participants with diabetes, by tuberculosis drug regimen, in the microbiologically eligible analysis population in a study assessing efficacy and safety of 4-month rifapentine-based tuberculosis treatments in persons with diabetes at sites in 12 countries (Brazil, Haiti, India, Kenya, Malawi, Peru, South Africa, Thailand, Uganda, United States, Vietnam, and Zimbabwe), January 2016–October 2018. Because scheduled study visits did not necessarily occur exactly at 8 weeks, the proportion of participants with culture conversion at 8 weeks is estimated from the Kaplan-Meier estimator at t = 10 weeks. Differences were not statistically significant for any comparisons.

We found no statistically significant difference in time to stable sputum culture conversion to negative in participants with diabetes treated with each of the experimental regimens compared with the control regimen: rifapentine/moxifloxacin regimen hazard ratio 1.3 (95% CI 0.9–1.9) in liquid media and 1.4 (95% CI 1.0–2.1) on solid media; rifapentine regimen hazard ratio 1.0 (95% CI 0.7–1.5) in liquid media and 1.1 (95% CI 0.8–1.7) on solid media (Figure 2). CIs were wide, and the point estimates of the hazard ratios were similar to those previously reported for the whole study population (*15*). In the microbiologically eligible population in participants with diabetes, culture conversion was achieved by the 8-week follow-up visit in liquid media in 62.3% in the control arm, 84.2% in the rifapentine/moxifloxacin arm, and 75.1% in the rifapentine arm and, on solid media, in 67.3% in the control arm, 91.2% in the rifapentine/moxifloxacin arm, and 85.6% in the rifapentine arm.

### Safety and Tolerability

Of 178 participants with diabetes included in the safety analysis population, 24.7% experienced grade >3 adverse events during treatment (31.6% in the control arm, 23.1% the in the rifapentine/moxifloxacin arm, and 19.6% in the rifapentine arm) (Table 2). The difference in proportion of participants with grade 3–5 adverse events between the control and rifapentine/moxifloxacin arm was –8.7% (95% CI –24.5 to 7.1), and the difference between the control and rifapentine arm was –11.0% (95% CI –26.7 to 4.8).

**Table 2:** Safety and tolerability among 178 participants with diabetes (safety analysis population*), by tuberculosis drug regimen, in a study assessing efficacy and safety of 4-month rifapentine-based tuberculosis treatments in persons with diabetes at sites in 12 countries,† January 2016–October 2018‡

Characteristic	Control, n = 57	Rifapentine/moxifloxacin, n = 65	Rifapentine, n = 56	Total, N = 178
Primary safety outcome				
Participants with grade >3 adverse event, no. (%)	18 (31.6)	15 (23.1)	11 (19.6)	44 (24.7)
Unadjusted risk difference compared with control (95% CI)		–8.7% (–24.5 to 7.1)	–11.0% (–26.7 to 4.8)	
Secondary safety outcome				
Participants with treatment-related grade >3 adverse event, no. (%)	4 (7.0)	7 (10.8)	4 (7.1)	15 (8.4)
Unadjusted risk difference compared with control (95% CI)		3.3% (–6.7 to13.2)	0.5% (–9.2 to 10.1)	
Other safety outcomes, no. (%)				
Participants with any serious adverse event during treatment	10 (17.5)	7 (10.8)	8 (14.3)	25 (14.0)
Participants who died§	2 (3.5)	0	0	2 (1.1)
Participants with any adverse event resulting in discontinuation of study treatment¶	0	4 (6.2)	2 (3.6)	6 (3.4)
Participants with any grade >3 adverse event during 28 weeks after randomization	18 (31.6)	19 (29.2)	13 (23.2)	50 (28.1)
Liver function test values, no. (%)				
ALT or AST >5-fold upper limit of normal#	2 (3.5)	4 (6.2)	2 (3.6)	8 (4.5)
ALT or AST >10-fold upper limit of normal	0	2 (3.1)	2 (3.6)	4 (2.2)
Serum total bilirubin ≥3-fold upper limit of normal**	1 (1.8)	5 (7.7)	3 (5.4)	9 (5.1)
ALT or AST >3-fold upper limit of normal plus serum total bilirubin >2-fold upper limit of normal (Hy’s Law)	1 (1.8)	3 (4.6)	2 (3.6)	6 (3.4)
Tolerability among microbiologically eligible analysis population, n = 166				
Discontinuation of assigned treatment for any reason, no. (%)	11/57 (19.3)	8/58 (13.8)	7/51 (13.7)	26/166 (15.7)
Unadjusted risk difference compared with control (95% CI)		–4.9 (–18.0 to 8.2)	–4.7 (–18.4 to 9.0)	

*The safety analysis population included all participants who underwent randomization and received >1 dose of the assigned treatment. Safety was assessed during the on-treatment period (the time during which the participants were receiving the study treatment and up to 14 days after the last dose), unless otherwise specified. Adverse events were graded by the site investigators on the basis of the Common Terminology Criteria for Adverse Events criteria, version 4.03 (*19*). †Brazil, Haiti, India, Kenya, Malawi, Peru, South Africa, Thailand, Uganda, United States, Vietnam, and Zimbabwe. ‡ALT, alanine aminotransferase; AST, aspartate aminotransferase. §In the control regimen group, 2 participants died from pulmonary tuberculosis. ¶In the rifapentine/moxifloxacin regimen group, 4 participants had hepatitis. In the rifapentine regimen group, 2 participants had hepatitis. #ALT or AST >5-fold upper limit of normal corresponds to grade >3. **Total bilirubin >3-fold upper limit of normal corresponds to grade >3.

Serious adverse events during treatment were experienced by 14% participants with diabetes (17.5% in the control arm, 10.8% in the rifapentine/moxifloxacin arm, and 14.3% in the rifapentine arm) (Table 2). Two deaths (3.5%) occurred in participants in the control arm and none in the rifapentine or rifapentine/moxifloxacin arms. Six participants permanently discontinued study treatment (6.2% the in the rifapentine/moxifloxacin arm and 3.6% in the rifapentine arm) (Table 2). The percentage of participants that had any transaminase value during treatment of >5-fold the upper limit of normal was highest in the rifapentine/moxifloxacin arm (6.2%, 4/65) compared with the rifapentine arm (3.6%, 2/56) and the control arm (3.5%, 2/57) (Table 2). No participants in the control regimen had any transaminase value of >10-fold the upper limit of the reference range, compared with 3.6% in the rifapentine arm and 3.1% in rifapentine/moxifloxacin arm. The most frequent adverse events among participants with diabetes were hepatitis (n = 14), hypertension (n = 9), and diabetes mellitus under inadequate control (n = 8) (Appendix Table 5). One case of peripheral neuropathy was reported in a participant in the rifapentine arm (Appendix Table 5).

Discontinuation of assigned treatment for any reason (tolerability) in microbiologically eligible analysis population was 19.3% in the control arm, 13.8% in the rifapentine/moxifloxacin arm, and 13.7% in the rifapentine arm (Table 2). The difference in proportion of discontinuation of assigned treatment for any reason between the control and rifapentine/moxifloxacin arm was –4.9% (95% CI –18.0% to 8.2%), and the difference between the control and rifapentine arm was –4.7% (95% CI –18.4% to 9.0%).

In a sensitivity safety analysis limited to participants with prior diabetes diagnosis, point estimates of grade >3 adverse events were higher than in analysis of all participants classified as having diabetes but showed similar findings across the regimens (Appendix Table 6). The proportion of participants with grade >3 adverse events was higher in participants with diabetes compared with those without diabetes (24.7% vs. 16.9%; p = 0.01).

### Pharmacokinetics

We compared model-estimated mean AUC_0–24h_ and C_max_ in participants classified with diabetes with those of participants without diabetes for each of the study drugs (Table 3). Rifamycin (rifampin and rifapentine) AUC_0–24h_ and C_max_ were similar among participants with diabetes and participants without diabetes. Participants with diabetes compared with participants without diabetes had lower AUC_0–24h_ values for moxifloxacin and ethambutol and higher C_max_ values for pyrazinamide, but the magnitude of these differences was modest.

**Table 3:** AUC_0–24h_ and C_max_ in participants with and without diabetes, by tuberculosis drug, in a study assessing efficacy and safety of 4-month rifapentine-based tuberculosis treatments in persons with diabetes at sites in 12 countries,* January 2016–October 2018†

Value	Diabetes status	No. participants	Mean	SD	p value‡
AUC_0–24h_, μg × h/mL					
Rifapentine	No	1,565	572.44	183.8	0.25
	Yes	122	553.98	169.1	
Moxifloxacin	No	783	25.51	7.0	0.0001
	Yes	66	22.34	6.0	
Rifampin	No	770	53.32	37.5	0.94
	Yes	59	53.69	35.2	
Isoniazid	No	2,335	16.52	12.1	0.51
	Yes	181	15.80	14.5	
Ethambutol	No	1,552	15.93	3.2	0.0002
	Yes	115	14.89	2.8	
Pyrazinamide	No	2,335	346.14	91.5	0.48
	Yes	181	340.77	99.2	
C_max_, μg/mL					
Rifapentine	No	1,565	33.10	8.7	0.17
	Yes	122	31.97	8.7	
Moxifloxacin	No	783	2.67	0.7	0.23
	Yes	66	2.55	0.8	
Rifampin	No	770	10.20	4.8	0.60
	Yes	59	10.52	4.5	
Isoniazid	No	2,335	2.83	0.9	0.25
	Yes	181	2.75	0.9	
Ethambutol	No	1,552	1.82	0.6	0.43
	Yes	115	1.87	0.6	
Pyrazinamide	No	2,335	30.34	7.2	0.008
	Yes	181	32.05	8.3	

*Brazil, Haiti, India, Kenya, Malawi, Peru, South Africa, Thailand, Uganda, United States, Vietnam, and Zimbabwe. †AUC_0–24h_, area under the concentration time curve from 0–24 hours; C_max_, maximal plasma concentration. ‡A t-test was used to compare pharmacokinetic parameters between participants classified as having or not having diabetes at enrollment.

## Discussion

In this prespecified subgroup analysis among participants with diabetes enrolled in the parent TB study, the efficacy of the 4-month rifapentine/moxifloxacin regimen was comparable to that of the control regimen: 13.8% (8/58) unfavorable outcomes in microbiologically eligible and 12.3% (7/57) unfavorable outcomes in assessable populations, among participants in the 4-month rifapentine/moxifloxacin regimen, compared with 26.3% (15/57) in microbiologically eligible and 17.6% (9/51) in assessable populations, for the control regimen. The 4-month rifapentine regimen without moxifloxacin had more unfavorable outcomes among participants with diabetes (29.4% [15/51]) compared with the control group (23.4% [11/47]). Thus, moxifloxacin was essential for the success of the 4-month regimen, including among persons with diabetes.

Participants with diabetes had higher overall proportion of unfavorable outcomes compared with participants without diabetes. The presence of severe TB disease, as indicated by baseline cavities on chest radiograph, was similar between participants with and without diabetes (72.9% vs 72.3%), although some indication of a higher bacillary load was observed in participants with diabetes at baseline because of shorter time-to-detection in liquid media. With regard to the study drug concentrations in participants with and without diabetes, rifamycin exposures unexpectedly were not different between persons with versus without diabetes, and differences in the pharmacokinetics of moxifloxacin, ethambutol, and pyrazinamide were modest. Of note, the proportion with unfavorable outcomes by arm was 14.6% in the control arm and 15.5% in the rifapentine/moxifloxacin arm in the overall study population (*15*) but 26.3% in the control and 13.8% in rifapentine/moxifloxacin arms among people with diabetes. The percentage of participants with TB-related unfavorable outcomes was 5.3% in the control and 3.5% in rifapentine/moxifloxacin arms. Those findings suggest that the high potency of the moxifloxacin and optimally dosed rifapentine in the experimental regimen might have played an important role in successful TB treatment in persons with diabetes.

Among participants with diabetes, both 4-month investigational rifapentine regimens appeared to have comparable (and perhaps even better) safety compared with the 6-month control regimen, including the proportion of participants with grade >3 adverse events, serious adverse events, and all-cause discontinuations. Mortality rates during TB treatment were low among participants with diabetes (1.1%), and no deaths were observed in the rifapentine/moxifloxacin and rifapentine arms. Mortality rates were also low in the overall study population (0.6%) (*15*). We did not observe an imbalance across study arms in diabetes-associated adverse events, such as diabetes mellitus under inadequate control, hyperglycemia, diabetic ketoacidosis, diabetic neuropathy, or diabetic retinopathy.

The percentage of participants with grade >3 adverse events was higher in participants with diabetes compared with those without diabetes (24.7% [44/178] vs. 16.9% (393/2,328). This increase might be attributable to age-related factors and underlying conditions in the diabetes population, given than participants with diabetes, compared with participants without diabetes, were older (median age 46 years vs. 33 years), and also might be attributable to diabetes-related adverse events, such as inadequate glucose control.

A limitation of our study is that testing for diabetes was only required at the time of enrollment, and not all study participants had HgbA1c tests done. Some participants were classified in this analysis to have diabetes solely on the basis of a laboratory test result (hemoglobin or random or fasting glucose test), and we recognize potential for transient hyperglycemia induced by acute illness (stress hyperglycemia) among patients with TB disease (*21*). However, we performed sensitivity analyses limited to participants with a prior established diabetes diagnosis, and efficacy and safety results were consistent. The parent trial was not powered for this subgroup analysis and had relatively few participants with diabetes (*18*). Thus, correspondingly large CIs around point estimates occurred for efficacy and safety outcomes. The prevalence of diabetes was relatively low (7.2%) among trial participants; however, it appears to be similar the comparative age-adjusted diabetes prevalence in the populations of Africa (5.3%) and general global populations (9.8%) (*22*). We noted an imbalance in numbers of participants with diabetes among the regimens; slightly more were randomized in the rifapentine/moxifloxacin arm, given that randomization was stratified by the site, cavitation, and HIV status at the baseline, but not by diabetes. Furthermore, because study protocol did not require blood glucose testing after enrollment, we could not assess the affect of glycemic control on TB treatment outcomes in participants with diabetes.

In conclusion, among participants in a larger TB treatment trial who had diabetes, we found the study’s rifapentine/moxifloxacin regimen had improved culture conversion on solid media and a numerically better point estimate for efficacy and similar safety to control. Further studies of TB treatment using the rifapentine/moxifloxacin regimen in larger numbers of patients with diabetes is needed. Our findings suggest that persons with diabetes are good candidates for the rifapentine/moxifloxacin regimen. 

Appendix Additional information about efficacy and safety of 4-month rifapentine-based tuberculosis treatments in persons with diabetes.
