# Supplementary material for: Efficacy and Safety of 4-Month Rifapentine-Based Tuberculosis Treatments in Persons with Diabetes
Source: Emerg Infect Dis. 2025 Mar;31(3):467–76. doi: 10.3201/eid3103.241634 (PMC11878306; doi:10.3201/eid3103.241634)
Supplement: Appendix — Additional information about efficacy and safety of 4-month rifapentine-based tuberculosis treatments in persons with diabetes. [file 24-1634-Techapp-s1.pdf]

Article DOI: https://doi.org/10.3201/eid3103.241634

EID cannot ensure accessibility for supplementary materials supplied by authors. Readers who have difficulty accessing supplementary content should contact the authors for assistance.

Efficacy and Safety of 4-Month Rifapentine-Based Tuberculosis Treatments in Persons with Diabetes

Appendix

Appendix Table 1. Characteristics of randomized trial participants with diabetes

Characteristic	Control, n = 59	Rifapentine-Moxifloxacin, n = 66	Rifapentine, n = 56	Total, N = 181
Male sex – no. (%)	36 (61.0)	44 (66.7)	42 (75.0)	122 (67.4)
Age, years – median (range)	46 (23–77)	47 (15–69)	46 (15–68)	46 (15–77)
Age group – no. (%)				
12–17 y	0 (0.0)	1 (1.5)	1 (1.8)	2 (1.1)
18–34 y	11 (18.6)	16 (24.2)	12 (21.4)	39 (21.5)
35 y and older	48 (81.4)	49 (74.2)	43 (76.8)	140 (77.3)
Race – no. (%) *				
Asian	13 (22.0)	20 (30.3)	13 (23.2)	46 (25.4)
Black or African American	28 (47.5)	26 (39.4)	23 (41.1)	77 (42.5)
White	6 (10.2)	6 (9.1)	3 (5.4)	15 (8.3)
More than one race	9 (15.3)	9 (13.6)	15 (26.8)	33 (18.2)
Other race	3 (5.1)	5 (7.6)	2 (3.6)	10 (5.5)
Hispanic Ethnicity- no. (%)	9 (15.3)	8 (12.1)	6 (10.7)	23 (12.7)
Geographic Region- no. (%)				
Africa	35 (59.3)	35 (53.0)	34 (60.7)	104 (57.5)
Asia	13 (22.0)	19 (28.8)	13 (23.2)	45 (24.9)
North America	5 (8.5)	6 (9.1)	4 (7.1)	15 (8.3)
South America	6 (10.2)	6 (9.1)	5 (8.9)	17 (9.4)
HIV-positive – no. (%)	5 (8.5)	5 (7.6)	6 (10.7)	16 (8.8)
Cavitation on baseline chest x-ray – no.(%)				
Absent	17 (28.8)	16 (24.2)	15 (26.8)	48 (26.5)
<4cm	20 (33.9)	31 (47.0)	25 (44.6)	76 (42.0)
≥4cm	22 (37.3)	19 (28.8)	16 (28.6)	57 (31.5)
Weight in kg – median (range)	57 (40–84)	56 (41–118)	56 (42–89)	56 (40–118)
Body mass index, kg/m^2 – median (range)	21 (14–34)	21 (16–39)	21 (15–35)	21 (14–39)
WHO smear grade				
Negative	5 (8.5)	2 (3.0)	4 (7.1)	11 (6.1)
Scanty or 1–9 AFB	8 (13.6)	15 (22.7)	9 (16.1)	32 (17.7)
1+	26 (44.1)	17 (25.8)	14 (25.0)	57 (31.5)
2+	8 (13.6)	15 (22.7)	16 (28.6)	39 (21.5)
3+	9 (15.3)	12 (18.2)	12 (21.4)	33 (18.2)
Positive (WHO Scale not used)	3 (5.1)	5 (7.6)	1 (1.8)	9 (5.0)
Current smoker – no. (%)	12 (20.3)	13 (19.7)	21 (37.5)	46 (25.4)
Diabetes type				
Type I	1 (1.7)	5 (7.6)	2 (3.6)	8 (4.4)
Type II	29 (49.2)	31 (47.0)	13 (23.2)	73 (40.3)
Unknown	1 (1.7)	0 (0.0)	1 (1.8)	2 (1.1)
Diabetes diagnosis not reported at baseline	28 (47.5)	30 (45.5)	40 (71.4)	98 (54.1)
HgbA1C, n, median (IQR) **	49, 9 (7–11)	49, 8 (7–11)	48, 7 (7–9)	146, 7 (7–11)
Participants reporting treatment with anti-diabetic drugs	22 (37.3)	29 (43.9)	11 (19.6)	62 (34.3)
Metformin (with or without insulin or other anti-diabetic drugs***)	6 (27.3)	10 (34.5)	5 (45.5)	21 (33.9)
Insulin (with or without metformin or other anti-diabetic drugs)	4 (18.2)	4 (13.8)	1 (9.1)	9 (14.5)

Characteristic	Control, n = 59	Rifapentine-Moxifloxacin, n = 66	Rifapentine, n = 56	Total, N = 181
Both metformin and insulin	1 (4.5)	5 (17.2)	3 (27.3)	9 (14.5)
Other anti-diabetic drugs (given either with Metformin or Insulin)	10 (45.5)	8 (27.6)	1 (9.1)	19 (30.6)
Other anti-diabetic drugs only	1 (4.5)	2 (6.9)	1 (9.1)	4 (6.5)

Note. *Race was self-reported by trial participants. ** HgbA1C levels were not available for 35 participants. *** Other anti-diabetic drugs are defined as glibenclamide, glimepiride, gliclazide, or glipizide. Abbreviations: kg, kilograms; m2, meters squared, WHO, World Health Organization.

Appendix Table 2. Characteristics of the participants with diabetes and without diabetes at baseline in S31/A5439 microbiologically eligible population

Characteristic	Participants with diabetes, N = 166	Participants without diabetes, N = 2177	Overall, N = 2343	P value
Male sex – no. (%)	111 (66.9)	1559 (71.6)	1670 (71.3)	0.19
Age, years – median (range)	46 (15–77)	30 (13–81)	30 (13–81)	<0.0001
Age group – no. (%)				<0.0001
12–17 y	2 (1.2)	61 (2.8)	63 (2.7)	
18–34 y	35 (21.1)	1339 (61.5)	1374 (58.6)	
35 y and older	129 (77.7)	777 (35.7)	906 (38.7)	
Race – no. (%) *				<0.0001
Asian	43 (25.9)	225 (10.3)	268 (11.4)	
Black or African American	70 (42.2)	1606 (73.8)	1676 (71.5)	
White	14 (8.4)	22 (1.0)	36 (1.5)	
More than one race	30 (18.1)	284 (13.0)	314 (13.4)	
Other race	9 (5.4)	35 (1.6)	44 (1.9)	
Not reported	0 (0.0)	5 (0.2)	5 (0.2)	
Hispanic Ethnicity- no. (%)	22 (13.3)	52 (2.4)	74 (3.2)	<0.0001
Geographic Region- no. (%)				<0.0001
Africa	94 (56.6)	1622 (74.5)	1716 (73.2)	
Asia	43 (25.9)	220 (10.1)	263 (11.2)	
North America	13 (7.8)	268 (12.3)	281 (12.0)	
South America	16 (9.6)	67 (3.1)	83 (3.5)	
HIV-positive – no. (%)	15 (9.0)	178 (8.2)	193 (8.2)	0.70
Cavitation on baseline chest XC-ray – no. (%)				0.005
Absent	43 (25.9)	582 (26.7)	625 (26.7)	
<4cm	74 (44.6)	700 (32.2)	774 (33.0)	
≥4cm	49 (29.5)	880 (40.4)	929 (39.7)	
Missing	0 (0.0)	15 (0.7)	15 (0.6)	
Weight in kg – median (range)	56 (40–118)	53 (40–122)	53 (40–122)	<0.0001
Body mass index, kg/m^2 – median (range)	21 (14–38)	19 (13–45)	19 (13–45)	0.95
WHO smear grade				<0.0001
Not Determined	0 (0.0)	3 (0.1)	3 (0.1)	
Negative	9 (5.4)	73 (3.4)	82 (3.5)	
Scanty or 1–9 AFB	30 (18.1)	367 (16.9)	397 (16.9)	
1+	53 (31.9)	476 (21.9)	529 (22.6)	
2+	35 (21.1)	650 (29.9)	685 (29.2)	
3+	31 (18.7)	590 (27.1)	621 (26.5)	
Positive (WHO Scale not used)	8 (4.8)	18 (0.8)	26 (1.1)	
Current smoker – no. (%)	39 (23.5)	502 (23.1)	541 (23.1)	0.02

Note. *Race was self-reported by trial participants. ** HgbA1C levels were not available for 35 participants. *** Other anti-diabetic drugs are defined as glibenclamide, glimepiride, gliclazide, or glipizide. Abbreviations: kg, kilograms; m2, meters squared, WHO, World Health Organization.

Appendix Table 3. Efficacy analysis among participants with diabetes in the microbiologically eligible and assessable analysis populations.

	Microbiologically Eligible Analysis Population				Assessable Analysis Population			
Characteristic	Control, n = 57	Rifapentine-Moxifloxacin, n = 58	Rifapentine, n = 51	Total, N = 166*	Control, n = 51	Rifapentine-Moxifloxacin, n = 57	Rifapentine, n = 47	Total, N = 155**
Favorable outcome - no. (%)	42 (73.7)	50 (86.2)	36 (70.6)	128 (77.1)	42 (82.4)	50 (87.7)	36 (76.6)	128 (82.6)
Culture negative status at month 12	39 (68.4)	49 (84.5)	35 (68.6)	123 (74.1)	39 (76.5)	49 (86.0)	35 (74.5)	123 (79.4)
Seen at month 12 but no sputum produced, or cultures contaminated or unevaluable	3 (5.3)	1 (1.7)	1 (2.0)	5 (3.0)	3 (5.9)	1 (1.8)	1 (2.1)	5 (3.2)
Unfavorable outcome - no. (%)	15 (26.3)	8 (13.8)	15 (29.4)	38 (22.9)	9 (17.6)	7 (12.3)	11 (23.4)	27 (17.4)
Tuberculosis-related	3 (5.3)	2 (3.4)	10 (19.6)	15 (9.0)	3 (5.9)	2 (3.5)	10 (21.3)	15 (9.7)
Two consecutive positive cultures at or after week 17	2 (3.5)	2 (3.4)	8 (15.7)	12 (7.2)	2 (3.9)	2 (3.5)	8 (17.0)	12 (7.7)
Not seen at month 12; last culture positive	1 (1.8)	0 (0.0)	1 (2.0)	2 (1.2)	1 (2.0)	0 (0.0)	1 (2.1)	2 (1.3)
Clinical diagnosis of tuberculosis recurrence and treatment restarted	0 (0.0)	0 (0.0)	1 (2.0)	1 (0.6)	0 (0.0)	0 (0.0)	1 (2.1)	1 (0.6)
Not tuberculosis-related	6 (10.5)	5 (8.6)	1 (2.0)	12 (7.2)	6 (11.8)	5 (8.8)	1 (2.1)	12 (7.7)
Consent withdrawn during treatment; no adverse event reported	3 (5.3)	1 (1.7)	0 (0.0)	4 (2.4)	3 (5.9)	1 (1.8)	0 (0.0)	4 (2.6)
Treatment changed due to adverse event	0 (0.0)	3 (5.2)	0 (0.0)	3 (1.8)	0 (0.0)	3 (5.3)	0 (0.0)	3 (1.9)
Death during treatment	2 (3.5)	0 (0.0)	0 (0.0)	2 (1.2)	2 (3.9)	0 (0.0)	0 (0.0)	2 (1.3)
Lost to follow-up during treatment	0 (0.0)	0 (0.0)	1 (2.0)	1 (0.6)	0 (0.0)	0 (0.0)	1 (2.1)	1 (0.6)
Consent withdrawn during treatment, after occurrence of adverse event	1 (1.8)	0 (0.0)	0 (0.0)	1 (0.6)	1 (2.0)	0 (0.0)	0 (0.0)	1 (0.6)
Treatment changed or restarted for other reasons	0 (0.0)	1 (1.7)	0 (0.0)	1 (0.6)	0 (0.0)	1 (1.8)	0 (0.0)	1 (0.6)
Not assessable - no. (%)	6 (10.5)	1 (1.7)	4 (7.8)	11 (6.6)	N/A	N/A	N/A	N/A
Not seen at month 12; last culture negative	4 (7.0)	1 (1.7)	4 (7.8)	9 (5.4)	N/A	N/A	N/A	N/A
Withdrawn from treatment due to pregnancy	2 (3.5)	0 (0.0)	0 (0.0)	2 (1.2)	N/A	N/A	N/A	N/A
Unadjusted Risk difference from control in % with unfavorable outcome (95% CI)	N/A	−12.5% (−27.0, 1.9)	3.1% (−13.8, 20.0)		N/A	−5.4% (- 18.9, 8.1)	5.8% (−10.2, 21.8)	

Note. *15 participants were excluded from the microbiologically eligible analysis population: 11 had baseline drug resistance, 3 had negative sputum cultures at baseline, and 1 was randomized in error. **11 participants were excluded from the assessable analysis population: 2 became pregnant, and 9 missed the month 12 visit with the last culture being negative. Abbreviations: N/A, not applicable.

Appendix Table 4. Sensitivity analysis: Efficacy analysis among participants with prior diabetes diagnosis, microbiologically eligible and assessable analysis populations*

	Microbiologically Eligible Analysis Population				Assessable Analysis Population			
Characteristic	Control, n = 31	Rifapentine-Moxifloxacin, n = 32	Rifapentine, n = 14	Total, N = 77	Control, n = 27	Rifapentine-Moxifloxacin, n = 31	Rifapentine, n = 14	Total, N = 72
Favorable outcome - no. (%)	22 (71.0)	26 (81.3)	8 (57.1)	56 (72.7)	22 (81.5)	26 (83.9)	8 (57.1)	56 (77.8)
Culture negative status at month 12	19 (61.3)	25 (78.1)	7 (50.0)	51 (66.2)	19 (70.4)	25 (80.6)	7 (50.0)	51 (70.8)
Seen at month 12 but no sputum produced, or cultures contaminated or unevaluable	3 (9.7)	1 (3.1)	1 (7.1)	5 (6.5)	3 (11.1)	1 (3.2)	1 (7.1)	5 (6.9)
Unfavorable outcome - no. (%)	9 (29.0)	6 (18.8)	6 (42.9)	21 (27.3)	5 (18.5)	5 (16.1)	6 (42.9)	16 (22.2)
Tuberculosis-related	2 (6.5)	1 (3.1)	6 (42.9)	9 (11.7)	2 (7.4)	1 (3.2)	6 (42.9)	9 (12.5)
Two consecutive positive cultures at or after week 17	1 (3.2)	1 (3.1)	4 (28.6)	6 (7.8)	1 (3.7)	1 (3.2)	4 (28.6)	6 (8.3)
Not seen at month 12; last culture positive	1 (3.2)	0 (0.0)	1 (7.1)	2 (2.6)	1 (3.7)	0 (0.0)	1 (7.1)	2 (2.8)
Clinical diagnosis of tuberculosis recurrence and treatment restarted	0 (0.0)	0 (0.0)	1 (7.1)	1 (1.3)	0 (0.0)	0 (0.0)	1 (7.1)	1 (1.4)
Not tuberculosis-related	3 (9.7)	4 (12.5)	0 (0.0)	7 (9.1)	3 (11.1)	4 (12.9)	0 (0.0)	7 (9.7)
Consent withdrawn during treatment; no adverse event reported	3 (9.7)	1 (3.1)	0 (0.0)	4 (5.2)	3 (11.1)	1 (3.2)	0 (0.0)	4 (5.6)
Treatment changed due to adverse event	0 (0.0)	3 (9.4)	0 (0.0)	3 (3.9)	0 (0.0)	3 (9.7)	0 (0.0)	3 (4.2)
Not assessable - no. (%)	4 (12.9)	1 (3.1)	0 (0.0)	5 (6.5)	N/A	N/A	N/A	N/A
Not seen at month 12; last culture negative	3 (9.7)	1 (3.1)	0 (0.0)	4 (5.2)	N/A	N/A	N/A	N/A
Withdrawn from treatment due to pregnancy	1 (3.2)	0 (0.0)	0 (0.0)	1 (1.3)	N/A	N/A	N/A	N/A
Unadjusted risk difference from control in % with unfavorable outcome (95% CI)		−9.9 (−31.0, 11.1)	13.1 (−17.1, 43.3)			−2.0 (−21.81, 17.81)	24.1 (−5.6, 53.8)	

Appendix Table 5. Primary safety outcome: grade 3 or higher adverse events during treatment (+14 d) by MedDRA preferred term among participants with diabetes (safety population)

MedDRA preferred term	Control, n = 57	Rifapentine-Moxifloxacin, n = 65	Rifapentine, n = 56	Total, N = 178
Hepatitis	3	8	3	14
Hypertension	5	3	1	9
Diabetes mellitus inadequate control	3	3	2	8
Hemoptysis	1	1	1	3
Hyperglycaemia	1	1	1	3
Pregnancy	2	0	0	2
Anaemia	1	0	0	1
Angioedema	0	0	1	1
Bladder transitional cell carcinoma	0	0	1	1
Bronchiectasis	0	1	0	1
Bronchospasm	0	1	0	1
Diabetes mellitus	1	0	0	1
Diabetic ketoacidosis	0	1	0	1
Diabetic neuropathy	0	1	0	1
Diabetic retinopathy	1	0	0	1
Gastroenteritis	1	0	0	1
Guillain-Barre syndrome	0	1	0	1
Hypochromic anaemia	1	0	0	1
Large intestine polyp	0	1	0	1
Limb injury	1	0	0	1
Neuropathy peripheral	0	0	1	1
Orchitis	0	0	1	1
Overdose	0	0	1	1
Pneumonia	1	0	0	1
Psoriasis	0	1	0	1
Pulmonary embolism	1	0	0	1
Pyrexia	0	1	0	1
Rash maculo-papular	0	1	0	1
Sepsis	1	0	0	1
Upper gastrointestinal hemorrhage	1	0	0	1
Vomiting	0	0	1	1
Total adverse events	25	25	14	64
Participants with any grade 3–5 adverse event	18 (31.6)	15 (23.1)	11 (19.6)	44 (24.7)

Note. Some participants had more than one grade 3 or higher adverse event.
Abbreviations: MedDRA, Medical Dictionary for Regulatory Activities. AE, adverse event.

Appendix Table 6. Sensitivity analysis: Safety and tolerability among participants with prior diabetes diagnosis (safety population)*

Characteristic	Control, n = 30	Rifapentine-Moxifloxacin, n = 35	Rifapentine, n = 16	Total, N = 81
Primary safety outcome - - no. (%)				
Participants with Grade 3 or higher adverse event	11 (36.7)	10 (28.6)	5 (31.3)	26 (32.1)
Difference in percentage (95% CI)†		−8.8 (−31.4, 13.8)	−5.8 (−31.7, 20.2)	
Secondary safety outcome - no. (%)				
Participants with treatment-related grade 3 or higher adverse event	2 (6.7)	5 (14.3)	3 (18.8)	10 (12.3)
Unadjusted risk difference compared with control (95% CI)		7.42 (−7.5, 22.3)	12.69 (−7.7, 33.1)	
Other safety outcomes - no. (%)				
Participants with any serious adverse events during treatment	7 (23.3)	4 (11.4)	4 (25.0)	15 (18.5)
Participants with death	0 (0.0)	0 (0.0)	0 (0.0)	0 (0.0)
Participants with any adverse event resulting in discontinuation of study treatment †	0 (0.0)	3 (8.6)	2 (12.5)	5 (6.2)
Participants with any grade 3 or higher adverse event during 28 weeks after randomization	11 (36.7)	11 (31.4)	5 (31.3)	27 (33.3)
ALT or AST ≥5-fold upper limit of normal [a]	0 (0.0)	3 (8.6)	1 (6.3)	4 (4.9)
ALT or AST ≥10-fold upper limit of normal	0 (0.0)	1 (2.9)	1 (6.3)	2 (2.5)
Serum total bilirubin ≥3-fold upper limit of normal [b]	1 (3.3)	3 (8.6)	2 (12.5)	6 (7.4)
ALT or AST ≥3-fold upper limit of normal plus serum total bilirubin ≥2-fold upper limit of normal (Hy's Law)	1 (3.3)	2 (5.7)	2 (12.5)	5 (6.2)
Tolerability (microbiologically eligible analysis population) - no. (%)				
Discontinuation of assigned treatment for any reason	6 (19.4)	6 (18.8)	2 (14.3)	14 (18.2)
Unadjusted risk difference compared with control (95% CI)†		−4.4 (−27.8, 18.9)	−0.02 (−19.7, 19.7)	

*Analysis population in this table includes participants with diabetes diagnoses reported at enrollment.
The safety analysis population included all the participants who had undergone randomization and received at least one dose of the assigned treatment. Safety was assessed during the on-treatment period (the time during which the participants were receiving the study treatment and up to 14 d after the last dose), unless otherwise specified. Adverse events were graded by the site investigators according to the National Cancer Institute Common Terminology Criteria for Adverse Events.
† Rifapentine-moxifloxacin regimen: 3 hepatitis. Rifapentine regimen: 2 hepatitis.
Abbreviations: ALT denotes alanine aminotransferase, AST aspartate aminotransferase, and ULN upper limit of the normal range.
[a]ALT or AST >5-fold upper limit of normal corresponds to Grade 3 or higher.
[b]Total bilirubin >3-fold upper limit of normal corresponds to Grade 3 or higher.

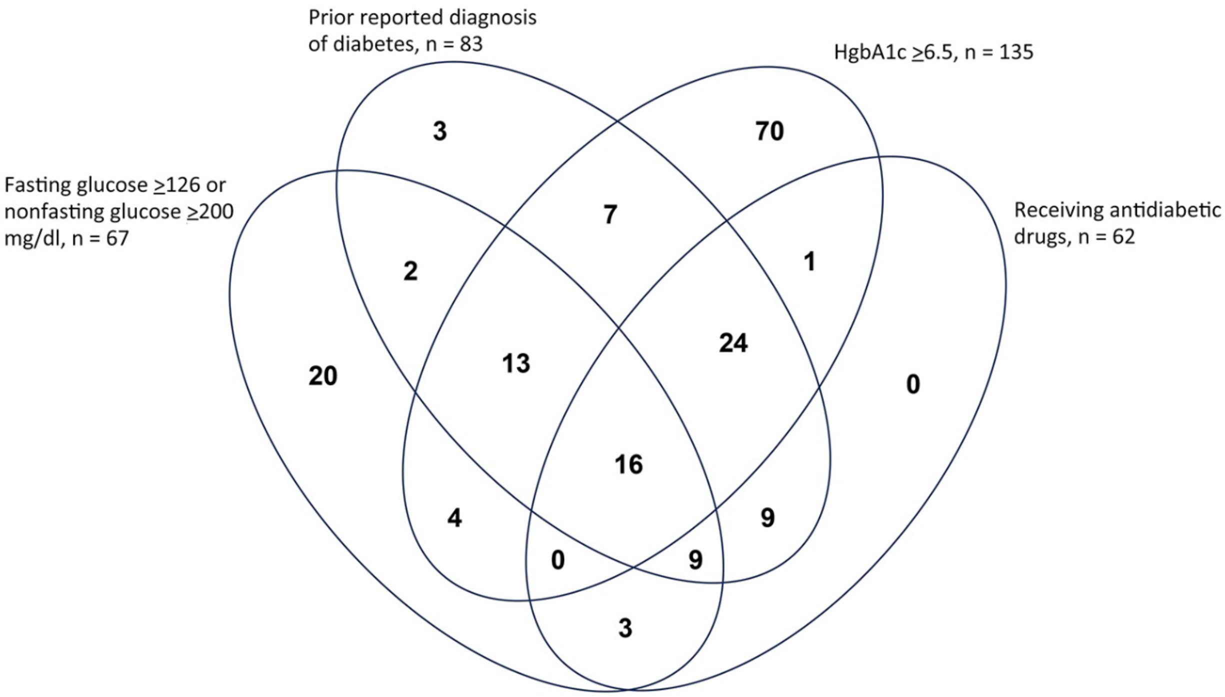

Appendix Figure. Diabetes criteria Venn diagram. Diabetes criteria were assessed at enrollment (baseline). At least one of these criteria was required to be included in these analyses. HbA1c is Hemoglobin A1c. Diabetes medications are WHO Drug Dictionary ACT Class 2 category “Drugs Used in Diabetes.”
